# Mechanisms Underlying the Essential Role of Mitochondrial Membrane Lipids in Yeast Chronological Aging

**DOI:** 10.1155/2017/2916985

**Published:** 2017-05-16

**Authors:** Younes Medkour, Paméla Dakik, Mélissa McAuley, Karamat Mohammad, Darya Mitrofanova, Vladimir I. Titorenko

**Affiliations:** Department of Biology, Concordia University, Montreal, QC, Canada H4B 1R6

## Abstract

The functional state of mitochondria is vital to cellular and organismal aging in eukaryotes across phyla. Studies in the yeast *Saccharomyces cerevisiae* have provided evidence that age-related changes in some aspects of mitochondrial functionality can create certain molecular signals. These signals can then define the rate of cellular aging by altering unidirectional and bidirectional communications between mitochondria and other organelles. Several aspects of mitochondrial functionality are known to impact the replicative and/or chronological modes of yeast aging. They include mitochondrial electron transport, membrane potential, reactive oxygen species, and protein synthesis and proteostasis, as well as mitochondrial synthesis of iron-sulfur clusters, amino acids, and NADPH. Our recent findings have revealed that the composition of mitochondrial membrane lipids is one of the key aspects of mitochondrial functionality affecting yeast chronological aging. We demonstrated that exogenously added lithocholic bile acid can delay chronological aging in yeast because it elicits specific changes in mitochondrial membrane lipids. These changes allow mitochondria to operate as signaling platforms that delay yeast chronological aging by orchestrating an institution and maintenance of a distinct cellular pattern. In this review, we discuss molecular and cellular mechanisms underlying the essential role of mitochondrial membrane lipids in yeast chronological aging.

## 1. Introduction

Mitochondria are indispensable for organismal physiology and health in all eukaryotes [1–9]. The efficiencies with which these organelles generate the bulk of cellular ATP and make biosynthetic intermediates for amino acids, nucleotides, and lipids are known to deteriorate with age [1, 3, 5, 9, 10]. Such age-related deterioration of mitochondrial functionality is the universal feature of aging in evolutionarily distant eukaryotic organisms [11].

Studies in *Saccharomyces cerevisiae* have uncovered several mechanisms underlying the essential role of mitochondria in the replicative and chronological modes of aging in this yeast [12–15]. Yeast replicative aging is assessed by measuring the maximum number of mitotic divisions that a mother cell can undergo before it enters a senescent state [16–18]. The replicative mode of yeast aging is likely to imitate not only aging of mitotically dividing human cells [16, 17, 19–22] but also aging of postmitotic tissues and organismal aging in nematode worms and humans [22–24]. Yeast chronological aging is evaluated by measuring the length of time during which a cell remains viable after becoming quiescent [12, 20, 25–27]. The chronological mode of yeast aging is believed to mimic aging of human cells that are temporarily or permanently unable to divide [20, 25, 26, 28–31]. It needs to be noted, however, that the chronological and replicative modes of yeast aging are likely to converge into a single aging process [12, 31–37].

Mechanisms underlying the essential roles of some traits of mitochondrial functionality in both modes of yeast aging have been recently reviewed [12–15, 20]. These traits in replicatively and chronologically aging yeast include mitochondrial electron transport chain and oxidative phosphorylation, membrane potential, reactive oxygen species (ROS) homeostasis, protein synthesis and proteostasis, iron-sulfur cluster formation, and synthesis of amino acids and NADPH [12–15, 20, 37–46].

Until recently, it was unknown if such trait of mitochondrial functionality as the composition of mitochondrial membrane lipids can influence aging in yeast. Our recent studies have revealed that lithocholic bile acid (LCA) can delay the onset and decrease the rate of yeast chronological aging [12, 13, 47–54]. We demonstrated that the robust geroprotective effect of exogenously added LCA is due to its ability to cause certain changes in lipid compositions of both mitochondrial membranes. These changes in mitochondrial membrane lipids enable mitochondria to establish and maintain an aging-delaying pattern of the entire cell. Here, we review mechanisms through which LCA-induced changes in the composition of mitochondrial membrane lipids trigger a multistep process of converting mitochondria into signaling platforms that orchestrate such distinct cellular pattern.

## 2. Some Aspects of the Maintenance of Lipid Homeostasis Are Essential for Healthy Aging in Eukaryotes across Phyla

Early studies in the nematode *Caenorhabditis elegans*, the fruit fly *Drosophila melanogaster*, and mice have revealed that an attenuation of the proaging insulin/insulin-like growth factor 1 signaling pathway extends organismal lifespan and causes the accumulation of storage lipids [55–61]. These studies have suggested a link between lipid metabolism and healthy aging. Recent findings provide strong evidence that certain pathways of lipid metabolism and transport define lifespan and healthspan in evolutionarily distant eukaryotes, including the yeast *S. cerevisiae*, the nematode *C. elegans*, the fruit fly *D. melanogaster*, mammals, and possibly humans.

### 2.1. The Yeast *S. cerevisiae*

In *S. cerevisiae* cells, the metabolic pathway for ceramide and sphingolipid synthesis is an essential node of a complex signaling network known to define replicative and chronological lifespans [14, 62–66]. Other nodes of this network include such nutrient-sensing signaling pathways and protein kinases at the proaging TORC1 and TORC2 (target of rapamycin complexes 1 and 2, resp.) pathways, the antiaging mitochondrial retrograde signaling pathway, the proaging PKA (protein kinase A) pathway, the proaging protein kinases Pkh1 and Pkh2, and the proaging protein kinases Sch9 and Ypk2 [14, 62–66]. The unidirectional and bidirectional flow of information between the ceramide/sphingolipid synthesis node and other nodes of the network defines the rate of yeast replicative and chronological aging because the network orchestrates numerous longevity-defining cellular processes [14, 62–66]. Among these downstream cellular processes are general autophagy and mitophagy, stress response, genomic stability maintenance, ribosomal protein and RNA synthesis, amino acid synthesis, carbon and energy metabolism, and mitochondrial respiration [14, 62–66].

Another aspect of lipid metabolism and transport known to define longevity of chronologically aging yeast is the abundance of triacylglycerols (TAGs) [67–70]. These so-called neutral lipids are synthesized in the endoplasmic reticulum (ER) and then deposited in lipid droplets (LDs) [71–73]. The age-related accumulation of TAGs in LDs is a longevity assurance process that extends yeast chronological lifespan independently of the network that integrates ceramide/sphingolipid synthesis with nutrient-sensing signaling pathways and protein kinases [69, 70]. TAGs may delay yeast chronological aging because their accumulation in LDs allows to deposit a bulk of unsaturated fatty acids by esterifying them into TAGs [69, 70]. Because unsaturated fatty acids exhibit high susceptibility to age-related oxidative damage, their deposition in the form of TAGs may make LDs the major target of such damage; this would alleviate oxidative damage to macromolecules in other cellular locations [69, 70]. In addition, the esterification of unsaturated fatty acids into TAGs may delay yeast chronological aging by attenuating an age-related form of liponecrotic cell death known to be elicited by these fatty acids [67, 68, 74–76].

### 2.2. The Nematode *C. elegans*

The extent of longevity extension by various genetic interventions in *C. elegans* has been shown to correlate with the following coordinated changes in the concentrations/activities of enzymes involved in fatty acid elongation and desaturation: (1) decreased concentrations/activities of elongases involved in the synthesis of very long-chain fatty acids, which are known to lower membrane fluidity; (2) increased concentrations/activities of Δ9 desaturases involved in the formation of oxidation-resistant monounsaturated fatty acids (MUFAs); and (3) lowered concentration/activity of a Δ5 desaturase involved in the formation of oxidation-sensitive polyunsaturated fatty acids (PUFAs) [77–82]. The resulting decline in fatty-acid chain length, increase in MUFA concentrations, and decrease in PUFA concentrations are believed to constitute a “signature” of extended longevity and delayed aging in this nematode [77, 79, 81, 82]. This is likely because the establishment and maintenance of such prolongevity pattern of fatty acid composition allow to delay aging by enabling to sustain membrane fluidity and increase oxidative stress resistance [77, 80, 81].

Furthermore, an activation of the lipolysis of TAGs delays nematode aging because it increases the concentration of arachidonic acid, a polyunsaturated omega-6 fatty acid known to stimulate the prolongevity process of autophagy [81–86]. Nematode aging can also be decelerated by the accumulation of TAGs, either in a certain tissue and at a distinct stage of development or in response to some diets. Such aging-delaying accumulation of TAGs has been reported under the following conditions: (1) upon entry into dauer, due to an LKB1/AMPK- (liver kinase B1/AMP-activated protein kinase-) driven inhibition of TAG lipolysis in the adipose-like hypodermis tissue [87]; and (2) in response to nutrient-rich food, due to a mutation in the Rictor protein component of TORC2 [88].

In addition, some lipid classes have been shown to delay aging in *C. elegans* because they act as signaling molecules that can establish and maintain a prolongevity transcription pattern; these lipid classes include the bile acid-like steroids called dafachronic acids of germ-line ablated nematode mutants as well as the *N*-acylethanolamine fatty acid derivative called oleoylethanolamide [80, 89–95].

Moreover, an attenuation of mitochondrial proteostasis in *C. elegans* is known to activate the mitochondrial unfolded protein response (UPR^mt^) [96, 97]. UPR^mt^ has been shown to delay nematode aging in part because it elicits a global remodeling of lipid metabolism, which includes the accumulation of cardiolipins and fatty acids [98]. This global remodeling of lipid metabolism allows to turn on a so-called mitochondrial-to-cytosolic stress response, thereby enabling to maintain the prolongevity process of cytosolic protein homeostasis [98].

### 2.3. The Fruit Fly *D. melanogaster*

The accumulation of TAGs in fruit fly mutants deficient in LD-associated TAG lipase Brummer has been shown to delay fruit fly aging only under starvation conditions [99]. Furthermore, the macrocyclic lactone rapamycin is known to delay aging and increase the concentration of TAGs in fruit flies [100]; it remains to be seen, however, if such rapamycin-driven rise in TAG concentration in fruit flies has a causal role in the aging-delaying effect of this macrocyclic lactone.

### 2.4. Mammals and Humans

Aging of laboratory mice can be delayed in response to a decrease in the concentration of TAGs in white adipose tissue (WAT), which can be achieved either by genetically eliminating the WAT-specific insulin receptor [101] or by replacing the C/EBP*α* (CCAAT/enhancer-binding protein *α*) protein with its paralogue C/EBP*β* [102].

Moreover, the sirtuin SIRT1 has been shown to repress transcription of nuclear genes needed for the synthesis of TAGs in WAT of laboratory mice [103]. It has been proposed that the resulting decrease in TAG concentration in WAT is in part responsible for the delay of aging by caloric restriction, a robust longevity-extending dietary intervention known to increase the abundance of SIRT1 and also to activate the lipolytic degradation of TAGs in mice WAT [104].

The mass spectrometry-based identification and quantitation of numerous lipid classes have been recently used for comparative profiling of the plasma lipidomes and lipidomes of different tissues in long-lived and short-lived mammalian species, in ad libitum-fed mice and in mice placed on a CR diet, as well as in healthy human individuals with exceptional longevity and in their children. Such correlative profiling has revealed the following trends of a so-called “lipidomic signature” of extended longevity and delayed aging in mammals and humans: (1) a decreased extent of fatty acid unsaturation, which lowers both the double bond and peroxidizability indexes of different lipid classes; (2) declined concentrations of long-chain free fatty acids; (3) increased MUFA-to-PUFA ratio; (4) decreased levels of several sphingolipids, certain lysophosphatidylcholines and phosphatidylcholines, as well as highly polyunsaturated TAGs and diacylglycerols (DAGs); and (5) increased concentrations of some sphingomyelins and cholesteryl esters, as well as TAGs and DAGs with low extent of fatty acid unsaturation [81, 105–111]. Although the establishment of the key trends of this lipidomic signature is an essential first step towards defining lipid biomarkers of healthy aging and extended lifespan, it remains to be seen if any of the above trends has a causal mechanistic role in aging delay and longevity extension.

## 3. A Chemical Genetic Screen for Molecules That Delay Yeast Chronological Aging by Targeting Lipid Metabolism

Caloric restriction and dietary restriction (CR and DR, resp.) are two dietary interventions that slow aging and extend healthy lifespan in eukaryotes across phyla [112–117]. Aging and the onset of age-related disorders can also be delayed by some chemical compounds of plant and microbial origin. Among these geroprotective compounds are resveratrol, rapamycin, curcumin, fisetin, quercetin, caffeine, and spermidine [112, 114, 118–124]. All these geroprotectors delay aging and age-related disorders only under non-CR or non-DR conditions, that is, when the intake of calories or nutrients is not restricted [47, 112, 114, 120, 122, 125–129]. Moreover, all these geroprotective compounds of plant and microbial origin have been shown to modulate a signaling network integrating pathways and protein kinases that are under the stringent control of calorie or nutrient availability [47, 112, 114, 117, 120, 122, 128]. The term “adaptable” was therefore coined for these networks, pathways, and protein kinases [47]. We sought to find chemical geroprotectors that delay aging and age-related disorders under CR conditions by modulating a different kind of pathways, the ones that define longevity irrespective of calorie and nutrient supply. We call these longevity pathways “constitutive” or “housekeeping” [47]. Moreover, because of our interest in mechanisms through which lipids influence yeast chronological aging, we were looking for geroprotective small molecules that modulate “constitutive” or “housekeeping” longevity pathways by targeting lipid metabolism and transport [47]. We therefore conducted a high-throughput chemical genetic screen for small molecules that can extend the chronological lifespan of the single-gene-deletion mutant strain *pex5*Δ. The *pex5*Δ strain is impaired in peroxisomal oxidation of fatty acids and, hence, is unable to generate peroxisomal acetyl-CoA for ATP synthesis in mitochondria [47]. Our screen of numerous chemical compounds from several commercial libraries has identified 24 geroprotective small molecules that can slow chronological aging by remodeling lipid metabolism not only in *pex5*Δ but also in wild-type yeast cells [47]. One of these molecules, a bile acid called LCA, exhibited the highest delaying effect on the chronological aging of yeast cultured under CR conditions [47]. We showed that several other bile acids (including deoxycholic acid, chenodeoxycholic acid, dehydrocholic acid, and hyodeoxycholic acid) delay yeast chronological aging to a significantly lesser degree than LCA, which is the most hydrophobic bile acid [47].

## 4. The Distribution of Exogenously Added LCA within a Yeast Cell

Unlike animals and humans, yeast cells do not synthesize bile acids [130–133]. Thus, a mechanism through which exogenously added LCA delays yeast chronological aging may or may not involve its entry into the yeast cell and, perhaps, a delivery of this highly hydrophobic bile acid to some specific location(s) within the cell. To investigate this important aspect of aging delay by LCA, we assessed the spatial distribution of exogenously added LCA in the yeast cell using a combination of subcellular fractionation by differential centrifugation, equilibrium density gradient centrifugation, and quantitative mass spectrometry [50]. We found that exogenously added LCA crosses both the cell wall and the plasma membrane to enter the yeast cell [50]. Our studies also revealed that intracellular LCA is sorted exclusively to mitochondria [50]. To investigate the spatial distribution of LCA within the mitochondrion, we subjected purified mitochondria to fractionation using a swell-shrink procedure and subsequent equilibrium density gradient centrifugation; we also fractionated purified mitochondria with the help of sonication followed by differential centrifugation [50]. Our quantitative mass spectrometric analysis of LCA recovered in different mitochondrial subcompartments showed that the majority (up to 80%) of the mitochondria-confined pool of LCA associates with the inner mitochondrial membrane (IMM), whereas a minor portion (not more than 20%) of this bile acid resides in the outer mitochondrial membrane (OMM) [50].

## 5. LCA in Mitochondria Alters the Abundance and Relative Concentrations of Membrane Phospholipids

**Figure 1:**
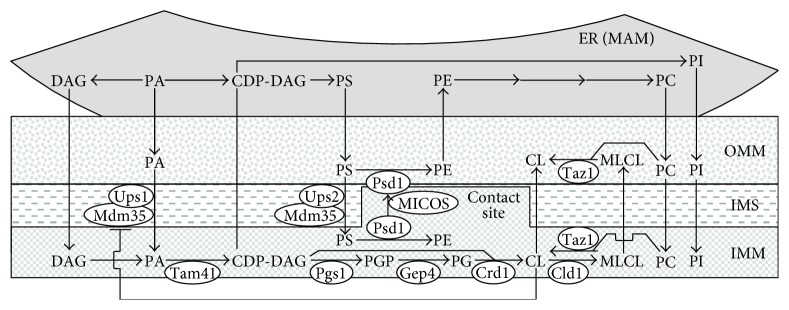
Processes that define the relative concentrations of different phospholipid classes in both membranes of yeast mitochondria. These processes are facilitated by proteins that reside in the IMM, IMS, OMM, and ER. Only proteins facilitating these processes in the IMM, IMS, and OMM are shown. A T bar denotes a CL-dependent inhibition of PA transfer from the OMM to the IMM. See text for more details. CDP-DAG: cytidine diphosphate-diacylglycerol; CL: cardiolipin; DAG: diacylglycerol; ER: endoplasmic reticulum; IMM: inner mitochondrial membrane; IMS: intermembrane space; MAM: mitochondria-associated membrane domain of the ER; MICOS: mitochondrial contact site protein complex; MLCL: monolysocardiolipin; OMM: outer mitochondrial membrane; PA: phosphatidic acid; PC: phosphatidylcholine; PG: phosphatidylglycerol; PGP: phosphatidylglycerol-phosphate; PI: phosphatidylinositol; PS: phosphatidylserine.

The relative concentrations of different membrane phospholipids in yeast mitochondria depend on a spatiotemporal dynamics of several processes that are facilitated by proteins residing in both mitochondria and the ER. These processes include the following: (1) the synthesis of phosphatidic acid (PA), cytidine diphosphate-diacylglycerol (CDP-DAG), diacylglycerol (DAG), phosphatidylserine (PS), phosphatidylcholine (PC), and phosphatidylinositol (PI) in the ER; (2) the conversion of ER-derived PA into CDP-DAG, phosphatidylglycerol-phosphate (PGP), phosphatidylglycerol (PG), cardiolipin (CL), and monolysocardiolipin (MLCL) in the IMM; (3) the transfer of PA from the ER to the OMM via mitochondria-ER contact sites, which are located at zones of close apposition between the OMM and the mitochondria-associated membrane (MAM) domain of the ER; (4) the movement of PA from the OMM via the intermembrane space (IMS) to the IMM, which is facilitated by the Ups1/Mdm35 protein complex; (5) the Ups2/Mdm35-dependent transfer of PS from the OMM across the IMS to the IMM, where PS serves as a substrate for PE synthesis catalyzed by Psd1; (6) the Psd1-dependent conversion of PS into PE in the OMM, which requires a juxtaposition of the two mitochondrial membranes and is facilitated by the mitochondrial contact site (MICOS) protein complex; (7) the movement of PC and PI from the ER to the OMM through mitochondria-ER contact sites, which is followed by PC and PI transfer from the OMM to the IMM via currently unknown mechanisms; (8) the transfer of DAG and CDP-DAG from the ER to the OMM through mitochondria-ER contact sites and the subsequent movement of these two phospholipids to the IMM by mechanisms that remain to be established; and (9) the CL-dependent inhibition of the Ups1/Mdm35-dependent transfer of PA from the OMM across the IMS to the IMM (Figure 1) [50, 134–137].

**Figure 2:**
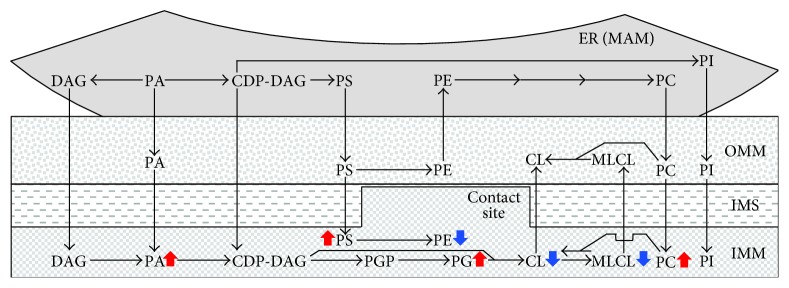
LCA exhibits differential effects on the relative concentrations of various phospholipid classes in mitochondrial membranes of yeast exposed to this bile acid. Arrows next to the names of individual phospholipids indicate phospholipid classes whose concentrations are increased (red arrows) or decreased (blue arrows) in cells cultured with exogenous LCA and therefore accumulating this bile acid in the IMM and OMM. See text for more details. Abbreviations are as provided in the legend for Figure 1.

Because LCA associates with the IMM and also resides in the OMM, we thought that this bile acid may modulate the spatiotemporal dynamics of phospholipid synthesis and transfer in yeast mitochondria and/or phospholipid movement from the ER to mitochondria [50]. We hypothesized that such LCA-driven modulation of phospholipid synthesis and transfer may, in turn, alter the abundance and/or relative concentrations of some classes of phospholipids in mitochondrial membranes [50]. To verify our hypothesis, we used quantitative mass spectrometry to compare mitochondrial lipidomes of yeast cultured under CR conditions with or without LCA. We found that LCA causes the following major changes in the abundance and composition of mitochondrial membrane phospholipids: (1) it elicits an age-related increase in the phospholipid/protein ratio of mitochondrial membranes and, thus, substantially elevates the abundance of membrane phospholipids in mitochondria; (2) it increases the relative concentrations of PA, PG, PS, and PC in mitochondrial membranes; and (3) it decreases the relative concentrations of CL, MLCL, and PE in mitochondrial membranes (Figure 2) [50].

Based on these data (which included data on the number of saturated and unsaturated acyl chains for each class of phospholipids), we calculated the relative concentrations of phospholipid classes exhibiting the nonbilayer forming shape of a cone or an inverted cone. We found that LCA decreases the relative concentrations of the nonbilayer forming classes of phospholipids, which include the following: (1) PC, PI, PS, and PG phospholipids carrying only saturated acyl chains; (2) PE phospholipids with one or two unsaturated acyl chains; and (3) PA, CL, and MLCL phospholipids carrying either only saturated acyl chains or from one to four unsaturated acyl chains [50]. These nonbilayer forming phospholipid classes are known to increase the extent of membrane curving for the IMM, thus raising the abundance of mitochondrial cristae (formed by the IMM) and mitochondrial contact cites (formed between the IMM and OMM) [50, 137–144].

We also calculated the relative concentrations of phospholipid classes having the bilayer forming shape of a cylinder. We found that LCA increases the relative concentrations of these bilayer forming phospholipid classes, which include PE phospholipids with only saturated acyl chains, as well as PC, PI, PS, and PG phospholipids with one or two unsaturated acyl chains [50]. These bilayer forming phospholipid classes are known to decrease the extent of membrane curving for the IMM, thereby (1) increasing the abundance of the IMM domains having “flat” bilayer conformation; (2) decreasing the abundance of the IMM domains exhibiting negative curvature typical of mitochondrial contact sites; and (3) decreasing the abundance of the IMM domains displaying positive curvature characteristic of mitochondrial cristae [50, 137–144].

## 6. LCA Causes Major Changes in Mitochondrial Abundance and Morphology

**Figure 3:**
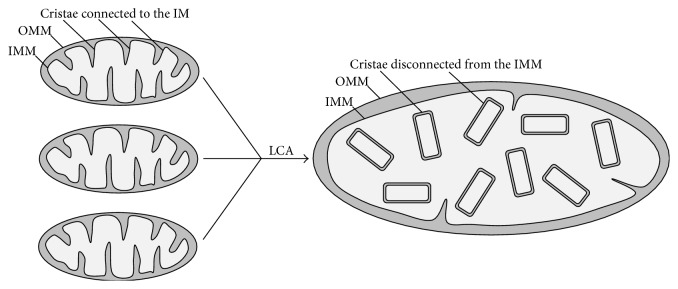
LCA increases mitochondrial size, reduces mitochondrial number, and elevates the abundance of mitochondrial cristae. Many mitochondrial cristae accumulate in the mitochondrial matrix because they are detached from the IMM. See text for more details. Abbreviations are as provided in the legend for Figure 1.

Because LCA markedly increases the abundance of mitochondrial membrane phospholipids (i.e., the ratio of phospholipid/protein in mitochondrial membranes; see above), we expected that this bile acid may elicit an expansion of both mitochondrial membranes to cause an enlargement of mitochondria. Our transmission electron microscopy (TEM) analysis has confirmed this expectation by revealing a significantly increased mitochondrial size in yeast cultured with LCA (Figure 3) [50, 51].

Because LCA increases the relative concentrations of PA, which is known for the ability to decrease mitochondrial number by promoting fusion of small mitochondria [145–150], we anticipated that this bile acid may decrease the number of mitochondria. As anticipated, TEM revealed a substantial decrease in mitochondrial number in yeast cultured with LCA (Figure 3) [50, 51].

LCA lessens the relative concentrations of the nonbilayer forming classes of phospholipids known to increase the extent of membrane curving for the IMM (see above); LCA also rises the relative concentrations of the bilayer forming classes of phospholipids shown to decrease the extent of membrane curving for the IMM (as described above). We therefore expected that LCA may increase the proportion of mitochondrial cristae detached from the IMM and having flat bilayer conformation and may also increase the abundance of these detached from the IMM cristae within the mitochondrial matrix. In support of these expectations, yeast cultured in the presence of LCA exhibited the following major morphological changes in the IMM and mitochondrial cristae: (1) many cristae were disconnected from the IMM and accumulated within the mitochondrial matrix in flat bilayer conformation and (2) the ratio of “total length of cristae/total length of the OMM” was significantly increased (Figure 3) [50, 51].

## 7. LCA Alters Mitochondrial Proteome

As described above, LCA causes major changes in the membrane lipidome, abundance, and morphology of mitochondria. We thought that these LCA-driven changes may affect mitochondrial protein import, folding, assembly, and other aspects of mitochondrial proteostasis, thereby altering mitochondrial proteome. In support of this notion, our quantitative mass spectrometric analysis revealed that LCA alters the age-related chronology of changes in the concentrations of many mitochondrial proteins known for their essential roles in some key mitochondrial functions [53, 54]. Among these mitochondrial functions are the tricarboxylic acid (TCA) cycle, glyoxylate cycle, electron transport chain (ETC), amino acid synthesis, heme synthesis and attachment, iron-sulfur cluster synthesis and assembly, NADPH synthesis, ROS detoxification, protein import and folding, stress response and protection, mitochondrial division, mitochondrial DNA replication and maintenance, and synthesis and translation of mitochondrial RNA [53]. Our bioinformatic analyses of how LCA alters the age-related chronology of changes in the concentrations of these various mitochondrial proteins demonstrated that they belong to two regulons called a partial mitochondrial dysfunction (PMD) regulon and an oxidative stress (OS) regulon, each regulated in response to a different aspect of limited mitochondrial function (Table 1) [53, 54]. Mitochondrial proteins that belong to PMD and OS regulons exhibit three different patterns of how their concentrations change with the chronological age of yeast cultured with LCA. We call these patterns “regulon type 1,” “regulon type 2,” and “regulon type 3”; each of the three patterns represents a distinct way of increasing or decreasing concentrations of certain mitochondrial proteins in yeast cells that progress through diauxic (D), postdiauxic (PD), and stationary (ST) growth phases (Table 1) [53, 54]. These three patterns are displayed in Table 1 and discussed elsewhere [53]. We found that the PMD and OS regulons can be divided into six or four clusters, respectively, each modulated by a different kind of partial mitochondrial dysfunctionality that triggers a distinct cellular response mediated by a discrete set of transcription factors; these transcription factors include Rtg1/Rtg2/Rtg3, Sfp1, Aft1, Yap1, Msn2/Msn4, Skn7, and Hog1 (Table 1) [53, 54]. As discussed by Beach [53], the PMD regulons include the following clusters: (1) *rho^0^* (a cluster of genes whose transcription is induced in response to complete loss of mitochondrial DNA); (2) S1 (a cluster of genes whose transcription is activated in response to inhibition of mitochondrial translation); (3) general TCA cycle dysfunction; (4) *kgd1*Δ, *kgd2*Δ, or *lpd1*Δ (a mutation eliminating the subunit Kgd1, Kgd2, or Lpd1 of the mitochondrial alpha-ketoglutarate dehydrogenase complex); (5) *yme1*Δ *mdl1*Δ (mutations that simultaneously eliminate the mitochondrial i-AAA (ATPases associated with diverse cellular activities) protease Yme1 and the mitochondrial ABC (ATP-binding cassette) transporter Mdl1, both involved in peptide export from mitochondria); and (6) *afo1*Δ (a mutation eliminating the mitochondrial ribosomal protein Afo1 of the large subunit) (Table 1). The OS regulons include clusters governed by transcription factors Yap1, Msn2/Msn4, Skn7, and Hog1 (Table 1) (as discussed elsewhere [53]). It needs to be emphasized that each of the transcription factors orchestrating various PMD and OS regulons in yeast cultured with LCA plays an essential role in the LCA-driven delay of yeast chronological aging [53, 54].

## 8. LCA Modifies Key Aspects of Mitochondrial Functionality

Because LCA alters mitochondrial lipidome and proteome and also elicits major changes in mitochondrial abundance and morphology, we thought that this bile acid may affect mitochondrial functionality. In support of this notion, we found that LCA markedly modifies the age-related chronology of four mitochondrial processes known to define the rate of aging in eukaryotes across phyla [50, 51]. These processes include mitochondrial respiration, membrane potential maintenance, ROS homeostasis preservation, and ATP synthesis [50, 51]. We demonstrated that in chronologically “young,” nonquiescent yeast, LCA allows to sustain the capacities of mitochondrial respiration, membrane potential maintenance, and ROS homeostasis preservation at a critical threshold [12, 50, 51]. At such threshold chronologically “young” cells exposed to LCA develop an antiaging cellular pattern that increases yeast chronological lifespan. This is because the capacities of mitochondrial respiration, membrane potential maintenance, and ROS homeostasis in chronologically “young” cells have specific impacts on many longevity-defining cellular processes and features, including (1) the concentrations of trehalose and glycogen;( 2) the concentrations of neutral lipids and the abundance of LDs; (3) peroxisomal fatty acid oxidation; (4) the concentrations of free fatty acids and DAGs; (5) glycolysis and gluconeogenesis; (6) ethanol metabolism; (7) mitochondrial translation; (8) mitochondrial size and number; (9) mitochondrial network formation and fragmentation; and (10) oxidative stress resistance (as discussed elsewhere [12]). We also demonstrated that in chronologically “old,” quiescent yeast, LCA (1) allows to maintain cellular ROS at a sublethal, “hormetic” concentration (known to delay aging by activating a signaling network that makes yeast resistant to various stresses [5, 12, 13, 68, 151–159]) and (2) increases the efficiencies of mitochondrial respiration, membrane potential maintenance, and ATP synthesis [50, 51].

## 9. LCA Elicits Age-Related Changes in the Concentrations of Many Proteins Located outside of Mitochondria

**Figure 4:**
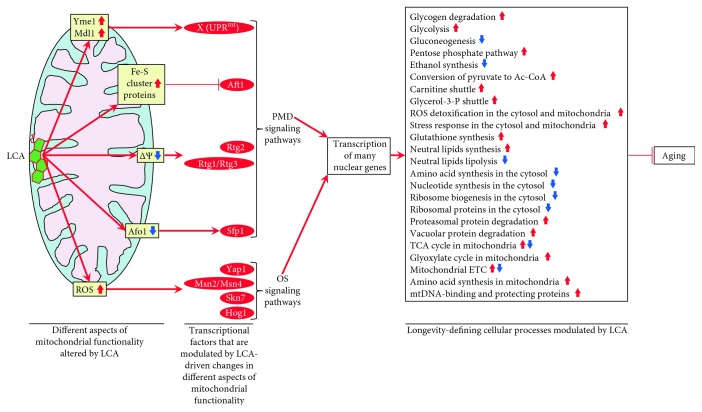
A model for how LCA-driven changes in different aspects of mitochondrial functionality modulate activities of a discrete set of transcription factors that are integrated into the partial mitochondrial dysfunction (PMD) and oxidative stress (OS) signaling pathways. These factors then orchestrate an establishment of an antiaging transcriptional program for numerous nuclear genes. These genes encode various cellular proteins that play essential roles in regulating longevity of chronologically aging yeast. See text for more details. Δ*Ψ*: the mitochondrial membrane potential. Other abbreviations are provided in the legend for Table 1.

The Rtg1/Rtg2/Rtg3, Sfp1, Aft1, Yap1, Msn2/Msn4, Skn7, and Hog1 proteins and protein complexes regulate transcription of nuclear genes that encode not only mitochondrial proteins (see above) but also numerous proteins in different cellular locations outside of mitochondria [160]. Therefore, we expected that LCA may cause major changes not only to mitochondrial proteome but also to the entire cellular proteome. As expected, our quantitative mass spectrometric analysis revealed that LCA alters the age-related chronology of changes in the concentrations of various proteins located outside of mitochondria [53, 54]. These proteins play essential roles in the glycolytic and pentose phosphate pathways, gluconeogenesis, glycogen degradation, ethanol formation, pyruvate conversion to acetyl-CoA, carnitine and glycerol-3-phosphate shuttling for maintaining NAD/NADH redox balance, neutral lipid synthesis and lipolysis, amino acid and nucleotide synthesis, glutathione synthesis, ROS decomposition, ribosome assembly, oxidative stress response, and proteasomal and vacuolar protein degradation (Figure 4) [53, 54]. Akin to mitochondrial proteins whose age-related expression pattern is driven by LCA and orchestrated by the above transcription factors, these nonmitochondrial proteins (1) belong to the multiclustered PMD and OS regulons; (2) exhibit three different patterns of increasing or decreasing concentrations of certain proteins in yeast progressing through D, PD, and ST growth phases; (3) are modulated in response to different kinds of partial mitochondrial dysfunctionality; and (4) are expressed under the control of a discrete set of transcription factors, including Rtg1/Rtg2/Rtg3, Sfp1, Aft1, Yap1, Msn2/Msn4, Skn7, and Hog1 [53, 54].

Based on these observations, we proposed a hypothetical model for how LCA-driven changes in different kinds of mitochondrial functionality modulate activities of the above transcription factors, all of which are integrated into the PMD and OS signaling pathways. This model is schematically depicted in Figure 4 and thoroughly discussed elsewhere [53]. In brief, we found that LCA elicits the following changes in mitochondrial functionality: (1) in chronologically “old” yeast, it increases cellular ROS and allows to maintain ROS at a sublethal, “hormetic” concentration; (2) in chronologically “old” yeast, it decreases the concentrations of several protein components of the large and small subunits of mitochondrial ribosome; (3) in chronologically “young” yeast, it lowers the mitochondrial membrane potential (Δ*Ψ*) and allows to sustain Δ*Ψ* at a critical threshold; (4) in chronologically “old” yeast, it increases the concentrations of mitochondrial proteins involved in the synthesis and assembly of iron-sulfur clusters, inorganic cofactors of many mitochondrial, nuclear, and cytosolic proteins playing essential roles in vital cellular processes; and (5) in chronologically “young” and “old” yeast, it increases the concentrations of mitochondrial and nonmitochondrial proteins known to be upregulated in response to a simultaneous lack of the mitochondrial i-AAA protease Yme1 and the mitochondrial ABC-transporter Mdl1 involved in peptide export from mitochondria (Figure 4) [53, 54]. These LCA-elicited changes in mitochondrial functionality alter activities of transcription factors Rtg1/Rtg2/Rtg3, Sfp1, Aft1, Yap1, Msn2/Msn4, Skn7, and Hog1 (Figure 4) [53, 54]. These transcription factors then trigger an antiaging transcriptional program for numerous nuclear genes, which encode cellular proteins implicated in oxidative stress response, proteostasis, lipid metabolism, and other longevity assurance processes taking place in chronologically aging yeast (Figure 4) [53, 54].

## 10. Conclusions

**Figure 5:**
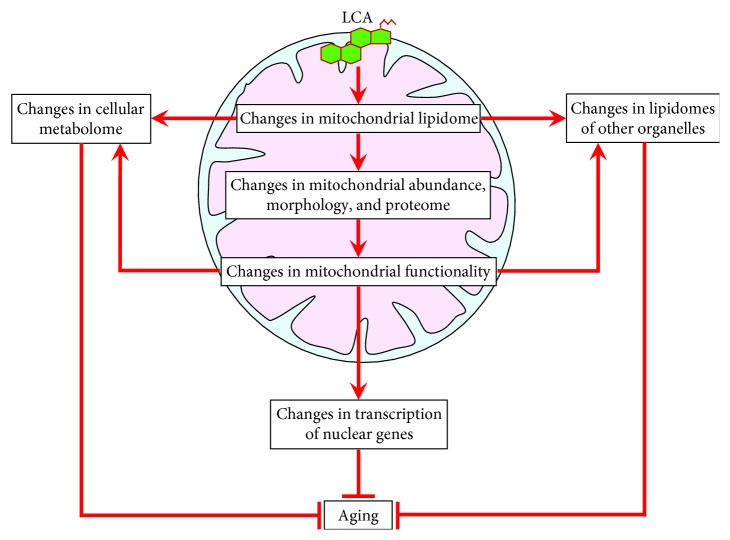
LCA causes specific changes in the relative concentrations of different classes of membrane lipids in mitochondria of chronologically aging yeast. Such LCA-dependent remodeling of mitochondrial lipidome triggers a cascade of consecutive events that establish an aging-delaying cellular pattern. See text for more details.

Our findings provide evidence that LCA delays yeast chronological aging by eliciting a remodeling of mitochondrial lipidome to cause specific changes in the relative concentrations of different classes of membrane lipids (Figure 5). This LCA-driven remodeling of mitochondrial lipidome triggers major changes in mitochondrial abundance and morphology and also alters mitochondrial proteome (Figure 5). These changes in the abundance, morphology, and protein composition of mitochondria lead to specific alterations in mitochondrial functionality (Figure 5). Our recent unpublished data indicate that the LCA-dependent alterations in mitochondrial lipidome, proteome, and morphology can also elicit changes in lipidomes of other organelles and in concentrations of a specific set of water-soluble metabolites (Arlia-Ciommo et al., in preparation) (Figure 5). By sensing different aspects of mitochondrial functional state, a discrete set of ten transcription factors orchestrates a distinct transcriptional program for many nuclear genes (Figure 5). The denouement of this cascade of consecutive events is the establishment of a cellular pattern that delays the onset and slows the progression of yeast chronological aging.

Of note, the proposed mechanism here for how the LCA-dependent remodeling of mitochondrial lipidome in the yeast *S. cerevisiae* allows to establish an aging-delaying cellular pattern is reminiscent of the mechanism in which the UPR^mt^-driven remodeling of mitochondrial lipidome in the nematode *C. elegans* triggers a cascade of events that institute an aging-delaying cellular pattern [98]. Moreover, the essential role of mitochondrial lipid metabolism in defining the pace of yeast chronological aging further supports the notion that the vital role of lipid homeostasis in healthy aging has been conserved in eukaryotes across phyla, including the yeast *S. cerevisiae* [14, 62–70], the nematode *C. elegans* [77–95, 98], the fruit fly *D. melanogaster* [99, 100], mammals [81, 101–109], and possibly humans [81, 105, 106, 110, 111].

## Figures and Tables

**Table 1:** In yeast cells that progress through diauxic (D), postdiauxic (PD), and stationary (ST) growth phases, LCA elicits three different patterns of changes in concentrations of various mitochondrial proteins. These patterns are called “regulon type 1,” “regulon type 2,” and “regulon type 3.” Every type of these regulons includes a partial mitochondrial dysfunction (PMD) regulon and an oxidative stress (OS) regulon, each regulated in response to a different aspect of limited mitochondrial function. The PMD and OS regulons are divided into six or four clusters, respectively, each modulated by a different kind of partial mitochondrial dysfunctionality that triggers a distinct cellular response mediated by a distinct set of transcription factors. These transcription factors include Rtg1/Rtg2/Rtg3, Sfp1, Aft1, Yap1, Msn2/Msn4, Skn7, and Hog1. See text for more details. *afo1*Δ: a mutation eliminating the mitochondrial ribosomal protein Afo1 of the large subunit; ETC: electron transport chain; *kgd1*Δ: a mutation eliminating the subunit Kgd1 of the mitochondrial alpha-ketoglutarate dehydrogenase complex; *kgd2*Δ: a mutation eliminating the subunit Kgd2 of the mitochondrial alpha-ketoglutarate dehydrogenase complex; *lpd1*Δ: a mutation eliminating the lipoamide dehydrogenase component Lpd1 of the pyruvate dehydrogenase and alpha-ketoglutarate dehydrogenase complexes; *mdl1*Δ: a mutation eliminating the mitochondrial ABC transporter Mdl1; *rho^0^*: a cluster of genes whose transcription is induced in response to complete loss of mitochondrial DNA; ROS: reactive oxygen species; S1: a cluster of genes whose transcription is activated in response to inhibition of mitochondrial translation; TCA: tricarboxylic acid cycle; *yme1*Δ: a mutation eliminating the catalytic subunit Yme1 of the mitochondrial i-AAA protease complex.

Pattern of age-related concentration change	Regulon	Cluster	Transcription factor(s)	Mitochondrial functions affected
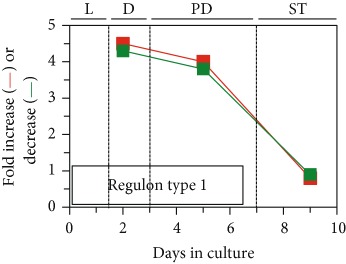	Partial mitochondrial dysfunction (PMD) regulon 1	*rho^0^*	Rtg 1/Rtg2/Rtg3, Sfp1, Aft1	TCA cycle, glyoxylate cycle, ETC, amino acid synthesis, heme synthesis and attachment, protein synthesis, protein import, protein folding and proteostasis, glycerol degradation, acetaldehyde metabolism
		S1	Rtg 1/Rtg2/Rtg3, Sfp1, Aft1	
		General TCA cycle dysfunction	Rtg 1/Rtg2/Rtg3, Sfp1, Aft1	
		*kgdl*Δ, *kgd2*Δ or *lpd1*Δ	Rtg 1/Rtg2/Rtg3, Sfp1, Aft1	
		*ymel*Δ *mdll*Δ	?	
		*afol*Δ	Sfp1	
	Oxidative stress (OS) regulon 1	Yap1 governed	Yap1	TCA cycle, glyoxylate cycle, ETC, amino acid synthesis, protein synthesis, protein import, protein folding and proteostasis, mtDNA replication and maintenance
		Msn2/Msn4 governed	Msn2/Msn4	
		Skn7 governed	Skn7	
		Hog1 governed	Hog1	

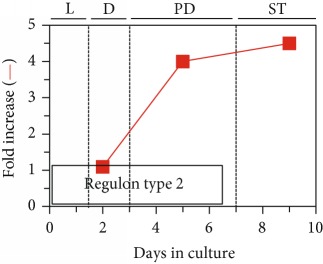	PMD regulon 2	*rho^0^*	Rtg 1/Rtg2/Rtg3, Sfp1, Aft1	Protein import, protein folding, and proteostasis
		S1	Rtg 1/Rtg2/Rtg3, Sfp1, Aft1	
		General TCA cycle dysfunction	Rtg 1/Rtg2/Rtg3, Sfp1, Aft1	
		*kgdl*Δ, *kgd2*Δ or *Ipdl*Δ	Rtg 1/Rtg2/Rtg3, Sfp1, Aft1	
		*yme1*Δ *mdl1*Δ	?	
		*afol*Δ	Sfp1	
	OS regulon 2	Yap1 governed	Yap1	Protein import, protein folding and proteostasis, ROS detoxification, stress response and protection, iron-sulfur clusters synthesis and assembly
		Msn2/Msn4 governed	Msn2/Msn4	
		Skn7 governed	Skn7	
		Hog1 governed	Hog1	

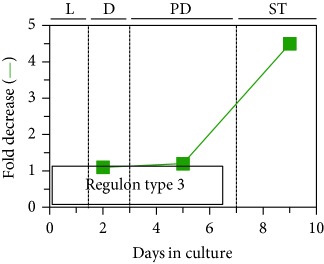	PMD regulon 3	*rho0*	Rtg1/Rtg2/Rtg3, Sfp1, Aft1	Mitochondrial division
		S1	Rtg1/Rtg2/Rtg3, Sfp1, Aft1	
		General TCA cycle dysfunction	Rtg1/Rtg2/Rtg3, Sfp1, Aft1	
		*kgd1*Δ, *kgd2*Δ or *lpd1*Δ	Rtg1/Rtg2/Rtg3, Sfp1, Aft1	
		*lpd1*Δ *yme1*Δ *mdl1*Δ	?	
		*afo1*Δ	Sfp1	
	OS regulon 3	Yap1 governed	Yap1	Mitochondrial division
		Msn2/Msn4 governed	Msn2/Msn4	
		Skn7 governed	Skn7	
		Hog1 governed	Hog1	

## References

[1] Nunnari J., Suomalainen A. (2012). Mitochondria: in sickness and in health. *Cell*.

[2] Boland M. L., Chourasia A. H., Macleod K. F. (2013). Mitochondrial dysfunction in cancer. *Frontiers in Oncology*.

[3] Pagliarini D. J., Rutter J. (2013). Hallmarks of a new era in mitochondrial biochemistry. *Genes and Development*.

[4] Chandel N. S. (2014). Mitochondria as signaling organelles. *BMC Biology*.

[5] Shadel G. S., Horvath T. L. (2015). Mitochondrial ROS signaling in organismal homeostasis. *Cell*.

[6] Mishra P., Chan D. C. (2016). Metabolic regulation of mitochondrial dynamics. *The Journal of Cell Biology*.

[7] Picard M., Wallace D. C., Burelle Y. (2016). The rise of mitochondria in medicine. *Mitochondrion*.

[8] Schrepfer E., Scorrano L. (2016). Mitofusins, from mitochondria to metabolism. *Molecular Cell*.

[9] Vyas S., Zaganjor E., Haigis M. C. (2016). Mitochondria and cancer. *Cell*.

[10] Vafai S. B., Mootha V. K. (2012). Mitochondrial disorders as windows into an ancient organelle. *Nature*.

[11] López-Otín C., Blasco M. A., Partridge L., Serrano M., Kroemer G. (2013). The hallmarks of aging. *Cell*.

[12] Arlia-Ciommo A., Leonov A., Piano A., Svistkova V., Titorenko V. I. (2014). Cell-autonomous mechanisms of chronological aging in the yeast *Saccharomyces cerevisiae*. *Microbial Cell*.

[13] Beach A., Leonov A., Arlia-Ciommo A., Svistkova V., Lutchman V., Titorenko V. I. (2015). Mechanisms by which different functional states of mitochondria define yeast longevity. *International Journal of Molecular Sciences*.

[14] Jazwinski S. M. (2015). Mitochondria to nucleus signaling and the role of ceramide in its integration into the suite of cell quality control processes during aging. *Ageing Research Reviews*.

[15] Ruetenik A., Barrientos A. (2015). Dietary restriction, mitochondrial function and aging: from yeast to humans. *Biochimica et Biophysica Acta*.

[16] Bitterman K. J., Medvedik O., Sinclair D. A. (2003). Longevity regulation in *Saccharomyces cerevisiae*: linking metabolism, genome stability, and heterochromatin. *Microbiology and Molecular Biology Reviews*.

[17] Steinkraus K. A., Kaeberlein M., Kennedy B. K. (2008). Replicative aging in yeast: the means to the end. *Annual Review of Cell and Developmental Biology*.

[18] Steffen K. K., Kennedy B. K., Kaeberlein M. (2009). Measuring replicative life span in the budding yeast. *Journal of Visualized Experiments*.

[19] Kaeberlein M. (2010). Lessons on longevity from budding yeast. *Nature*.

[20] Longo V. D., Shadel G. S., Kaeberlein M., Kennedy B. (2012). Replicative and chronological aging in *Saccharomyces cerevisiae*. *Cell Metabolism*.

[21] Denoth Lippuner A., Julou T., Barral Y. (2014). Budding yeast as a model organism to study the effects of age. *FEMS Microbiology Reviews*.

[22] McCormick M. A., Delaney J. R., Tsuchiya M. (2015). A comprehensive analysis of replicative lifespan in 4,698 single-gene deletion strains uncovers conserved mechanisms of aging. *Cell Metabolism*.

[23] Ghavidel A., Baxi K., Ignatchenko V. (2015). A genome scale screen for mutants with delayed exit from mitosis: Ire1-independent induction of autophagy integrates ER homeostasis into mitotic lifespan. *PLoS Genetics*.

[24] Janssens G. E., Veenhoff L. M. (2016). Evidence for the hallmarks of human aging in replicatively aging yeast. *Microbial Cell*.

[25] Fabrizio P., Longo V. D. (2007). The chronological life span of *Saccharomyces cerevisiae*. *Methods in Molecular Biology*.

[26] Longo V. D., Fabrizio P. (2012). Chronological aging in *Saccharomyces cerevisiae*. *Subcellular Biochemistry*.

[27] Piper P. W. (2012). Maximising the yeast chronological lifespan. *Subcellular Biochemistry*.

[28] Longo V. D., Kennedy B. K. (2006). Sirtuins in aging and age-related disease. *Cell*.

[29] Burtner C. R., Murakami C. J., Kennedy B. K., Kaeberlein M. (2009). A molecular mechanism of chronological aging in yeast. *Cell Cycle*.

[30] Burtner C. R., Murakami C. J., Olsen B., Kennedy B. K., Kaeberlein M. (2011). A genomic analysis of chronological longevity factors in budding yeast. *Cell Cycle*.

[31] Arlia-Ciommo A., Piano A., Leonov A., Svistkova V., Titorenko V. I. (2014). Quasi- programmed aging of budding yeast: a trade-off between programmed processes of cell proliferation, differentiation, stress response, survival and death defines yeast lifespan. *Cell Cycle*.

[32] Mirisola M. G., Longo V. D. (2012). Acetic acid and acidification accelerate chronological and replicative aging in yeast. *Cell Cycle*.

[33] Murakami C., Delaney J. R., Chou A. (2012). pH neutralization protects against reduction in replicative lifespan following chronological aging in yeast. *Cell Cycle*.

[34] Polymenis M., Kennedy B. K. (2012). Chronological and replicative lifespan in yeast: do they meet in the middle?. *Cell Cycle*.

[35] Delaney J. R., Murakami C., Chou A. (2013). Dietary restriction and mitochondrial function link replicative and chronological aging in *Saccharomyces cerevisiae*. *Experimental Gerontology*.

[36] Molon M., Zadrag-Tecza R., Bilinski T. (2015). The longevity in the yeast *Saccharomyces cerevisiae*: A comparison of two approaches for assessment the lifespan. *Biochemical and Biophysical Research Communications*.

[37] Dakik P., Titorenko V. I. (2016). Communications between mitochondria, the nucleus, vacuoles, peroxisomes, the endoplasmic reticulum, the plasma membrane, lipid droplets and the cytosol during yeast chronological aging. *Frontiers in Genetics*.

[38] Titorenko V. I., Terlecky S. R. (2011). Peroxisome metabolism and cellular aging. *Traffic*.

[39] Beach A., Burstein M. T., Richard V. R., Leonov A., Levy S., Titorenko V. I. (2012). Integration of peroxisomes into an endomembrane system that governs cellular aging. *Frontiers in Physiology*.

[40] Beach A., Titorenko V. I. (2013). Essential roles of peroxisomally produced and metabolized biomolecules in regulating yeast longevity. *Subcellular Biochemistry*.

[41] Leonov A., Titorenko V. I. (2013). A network of interorganellar communications underlies cellular aging. *International Union of Biochemistry and Molecular Biology Life*.

[42] da Cunha F. M., Torelli N. Q., Kowaltowski A. J. (2015). Mitochondrial retrograde signaling: triggers, pathways, and outcomes. *Oxidative Medicine and Cellular Longevity*.

[43] Eisenberg-Bord M., Schuldiner M. (2016). Ground control to major TOM: mitochondria-nucleus communication. *The FEBS Journal*.

[44] Perić M., Dib P. B., Dennerlein S. (2016). Crosstalk between cellular compartments protects against proteotoxicity and extends lifespan. *Scientific Reports*.

[45] Smethurst D. G., Cooper K. F. (2016). ER fatalities-The role of ER-mitochondrial contact sites in yeast life and death decisions. *Mechanisms of Ageing and Development*.

[46] Suhm T., Ott M. (2017). Mitochondrial translation and cellular stress response. *Cell and Tissue Research*.

[47] Goldberg A. A., Richard V. R., Kyryakov P. (2010). Chemical genetic screen identifies lithocholic acid as an anti-aging compound that extends yeast chronological life span in a TOR-independent manner, by modulating housekeeping longevity assurance processes. *Aging (Albany NY)*.

[48] Burstein M. T., Kyryakov P., Beach A. (2012). Lithocholic acid extends longevity of chronologically aging yeast only if added at certain critical periods of their lifespan. *Cell Cycle*.

[49] Richard V. R., Leonov A., Beach A. (2013). Macromitophagy is a longevity assurance process that in chronologically aging yeast limited in calorie supply sustains functional mitochondria and maintains cellular lipid homeostasis. *Aging (Albany NY)*.

[50] Beach A., Richard V. R., Leonov A. (2013). Mitochondrial membrane lipidome defines yeast longevity. *Aging (Albany NY)*.

[51] Burstein M. T., Titorenko V. I. (2014). A mitochondrially targeted compound delays aging in yeast through a mechanism linking mitochondrial membrane lipid metabolism to mitochondrial redox biology. *Redox Biology*.

[52] Arlia-Ciommo A., Piano A., Svistkova V., Mohtashami S., Titorenko V. I. (2014). Mechanisms underlying the anti-aging and anti-tumor effects of lithocholic bile acid. *International Journal of Molecular Sciences*.

[53] Beach A., Richard V. R., Bourque S. (2015). Lithocholic bile acid accumulated in yeast mitochondria orchestrates a development of an anti-aging cellular pattern by causing age-related changes in cellular proteome. *Cell Cycle*.

[54] Medkour Y., Titorenko V. I. (2016). Mitochondria operate as signaling platforms in yeast aging. *Aging (Albany NY)*.

[55] Kenyon C., Chang J., Gensch E., Rudner A., Tabtiang R. (1993). A *C. elegans* mutant that lives twice as long as wild type. *Nature*.

[56] Gems D., Sutton A. J., Sundermeyer M. L. (1998). Two pleiotropic classes of *daf-2* mutation affect larval arrest, adult behavior, reproduction and longevity in *Caenorhabditis elegans*. *Genetics*.

[57] Tissenbaum H. A., Ruvkun G. (1998). An insulin-like signaling pathway affects both longevity and reproduction in *Caenorhabditis elegans*. *Genetics*.

[58] Clancy D. J., Gems D., Harshman L. G. (2001). Extension of life-span by loss of CHICO, a *Drosophila* insulin receptor substrate protein. *Science*.

[59] Tatar M., Kopelman A., Epstein D., Tu M. P., Yin C. M., Garofalo R. S. (2001). A mutant *Drosophila* insulin receptor homolog that extends life-span and impairs neuroendocrine function. *Science*.

[60] Broughton S. J., Piper M. D., Ikeya T. (2005). Longer lifespan, altered metabolism, and stress resistance in Drosophila from ablation of cells making insulin-like ligands. *Proceedings of the National Academy of Sciences of the United States of America*.

[61] Yen K., Mobbs C. V. (2010). Evidence for only two independent pathways for decreasing senescence in *Caenorhabditis elegans*. *Age (Dordrecht, Netherlands)*.

[62] Huang X., Withers B. R., Dickson R. C. (2014). Sphingolipids and lifespan regulation. *Biochimica et Biophysica Acta*.

[63] Swinnen E., Ghillebert R., Wilms T., Winderickx J. (2014). Molecular mechanisms linking the evolutionary conserved TORC1-Sch9 nutrient signalling branch to lifespan regulation in *Saccharomyces cerevisiae*. *FEMS Yeast Research*.

[64] Swinnen E., Wilms T., Idkowiak-Baldys J. (2014). The protein kinase Sch9 is a key regulator of sphingolipid metabolism in *Saccharomyces cerevisiae*. *Molecular Biology of the Cell*.

[65] Eltschinger S., Loewith R. (2016). TOR complexes and the maintenance of cellular homeostasis. *Trends in Cell Biology*.

[66] Teixeira V., Costa V. (2016). Unraveling the role of the target of rapamycin signaling in sphingolipid metabolism. *Progress in Lipid Research*.

[67] Goldberg A. A., Bourque S. D., Kyryakov P. (2009). A novel function of lipid droplets in regulating longevity. *Biochemical Society Transactions*.

[68] Goldberg A. A., Bourque S. D., Kyryakov P. (2009). Effect of calorie restriction on the metabolic history of chronologically aging yeast. *Experimental Gerontology*.

[69] Handee W., Li X., Hall K. W. (2016). An energy-independent pro-longevity function of triacylglycerol in yeast. *PLoS Genetics*.

[70] Li X., Handee W., Kuo M. H. (2017). The slim, the fat, and the obese: guess who lives the longest?. *Current Genetics*.

[71] Walther T. C., Farese R. V. (2012). Lipid droplets and cellular lipid metabolism. *Annual Review of Biochemistry*.

[72] Kohlwein S. D., Veenhuis M., van der Klei I. J. (2013). Lipid droplets and peroxisomes: key players in cellular lipid homeostasis or a matter of fat - store 'em up or burn 'em down. *Genetics*.

[73] Pol A., Gross S. P., Parton R. G. (2014). Review: biogenesis of the multifunctional lipid droplet: lipids, proteins, and sites. *The Journal of Cell Biology*.

[74] Beach A., Titorenko V. I. (2011). In search of housekeeping pathways that regulate longevity. *Cell Cycle*.

[75] Sheibani S., Richard V. R., Beach A. (2014). Macromitophagy, neutral lipids synthesis and peroxisomal fatty acid oxidation protect yeast from “liponecrosis”, a previously unknown form of programmed cell death. *Cell Cycle*.

[76] Richard V. R., Beach A., Piano A. (2014). Mechanism of liponecrosis, a distinct mode of programmed cell death. *Cell Cycle*.

[77] Shmookler Reis R. J., Xu L., Lee H. (2011). Modulation of lipid biosynthesis contributes to stress resistance and longevity of *C. elegans* mutants. *Aging (Albany NY)*.

[78] Ackerman D., Gems D. (2012). The mystery of *C. elegans* aging: an emerging role for fat. Distant parallels between *C. elegans* aging and metabolic syndrome?. *BioEssays*.

[79] Hou N. S., Taubert S. (2012). Function and regulation of lipid biology in *Caenorhabditis elegans* aging. *Frontiers in Physiology*.

[80] Hansen M., Flatt T., Aguilaniu H. (2013). Reproduction, fat metabolism, and life span: what is the connection?. *Cell Metabolism*.

[81] Schroeder E. A., Brunet A. (2015). Lipid profiles and signals for long life. *Trends in Endocrinology and Metabolism*.

[82] Watts J. L. (2016). Using *Caenorhabditis elegans* to uncover conserved functions of omega-3 and omega-6 fatty acids. *Journal of Clinical Medicine*.

[83] Wang M. C., O'Rourke E. J., Ruvkun G. (2008). Fat metabolism links germline stem cells and longevity in *C. elegans*. *Science*.

[84] Kniazeva M., Han M. (2013). Fat chance for longevity. *Genes and Development*.

[85] O'Rourke E. J., Kuballa P., Xavier R., Ruvkun G. (2013). ω-6 Polyunsaturated fatty acids extend life span through the activation of autophagy. *Genes and Development*.

[86] Niso-Santano M., Malik S. A., Pietrocola F. (2015). Unsaturated fatty acids induce non-canonical autophagy. *The EMBO Journal*.

[87] Narbonne P., Roy R. (2009). *Caenorhabditis elegans* dauers need LKB1/AMPK to ration lipid reserves and ensure long-term survival. *Nature*.

[88] Soukas A. A., Kane E. A., Carr C. E., Melo J. A., Ruvkun G. (2009). Rictor/TORC2 regulates fat metabolism, feeding, growth, and life span in *Caenorhabditis elegans*. *Genes and Development*.

[89] Gerisch B., Rottiers V., Li D. (2007). A bile acid-like steroid modulates *Caenorhabditis elegans* lifespan through nuclear receptor signaling. *Proceedings of the National Academy of Sciences of the United States of America*.

[90] Antebi A. (2013). Regulation of longevity by the reproductive system. *Experimental Gerontology*.

[91] Magner D. B., Wollam J., Shen Y. (2013). The NHR-8 nuclear receptor regulates cholesterol and bile acid homeostasis in *C. elegans*. *Cell Metabolism*.

[92] Mahanti P., Bose N., Bethke A. (2014). Comparative metabolomics reveals endogenous ligands of DAF-12, a nuclear hormone receptor, regulating *C. elegans* development and lifespan. *Cell Metabolism*.

[93] Folick A., Oakley H. D., Yu Y. (2015). Aging. Lysosomal signaling molecules regulate longevity in *Caenorhabditis elegans*. *Science*.

[94] Aguilaniu H., Fabrizio P., Witting M. (2016). The role of dafachronic acid signaling in development and longevity in *Caenorhabditis elegans*: digging deeper using cutting-edge analytical chemistry. *Frontiers in Endocrinology*.

[95] Lemieux G. A., Ashrafi K. (2016). Investigating connections between metabolism, longevity, and behavior in *Caenorhabditis elegans*. *Trends in Endocrinology and Metabolism*.

[96] Haynes C. M., Fiorese C. J., Lin Y. F. (2013). Evaluating and responding to mitochondrial dysfunction: the mitochondrial unfolded-protein response and beyond. *Trends in Cell Biology*.

[97] Schulz A. M., Haynes C. M. (2015). UPR^mt^-mediated cytoprotection and organismal aging. *Biochimica et Biophysica Acta*.

[98] Kim H. E., Grant A. R., Simic M. S. (2016). Lipid biosynthesis coordinates a mitochondrial-to-cytosolic stress response. *Cell*.

[99] Grönke S., Mildner A., Fellert S. (2005). Brummer lipase is an evolutionary conserved fat storage regulator in *Drosophila*. *Cell Metabolism*.

[100] Bjedov I., Toivonen J. M., Kerr F. (2010). Mechanisms of life span extension by rapamycin in the fruit fly *Drosophila melanogaster*. *Cell Metabolism*.

[101] Blüher M., Kahn B. B., Kahn C. R. (2003). Extended longevity in mice lacking the insulin receptor in adipose tissue. *Science*.

[102] Chiu C. H., Lin W. D., Huang S. Y., Lee Y. H. (2004). Effect of a C/EBP gene replacement on mitochondrial biogenesis in fat cells. *Genes and Development*.

[103] Picard F., Kurtev M., Chung N., a. (2004). Sirt1 promotes fat mobilization in white adipocytes by repressing PPAR-gamma. *Nature*.

[104] Bordone L., Guarente L. (2005). Calorie restriction, SIRT1 and metabolism: understanding longevity. *Nature Reviews Molecular Cell Biology*.

[105] Gonzalez-Covarrubias V. (2013). Lipidomics in longevity and healthy aging. *Biogerontology*.

[106] Gonzalez-Covarrubias V., Beekman M., Uh H. W. (2013). Lipidomics of familial longevity. *Aging Cell*.

[107] Jové M., Naudí A., Aledo J. C. (2013). Plasma long-chain free fatty acids predict mammalian longevity. *Scientific Reports*.

[108] Naudí A., Jové M., Ayala V., Portero-Otín M., Barja G., Pamplona R. (2013). Membrane lipid unsaturation as physiological adaptation to animal longevity. *Frontiers in Physiology*.

[109] Jové M., Naudí A., Ramírez-Núñez O. (2014). Caloric restriction reveals a metabolomic and lipidomic signature in liver of male mice. *Aging Cell*.

[110] Mapstone M., Cheema A. K., Fiandaca M. S. (2014). Plasma phospholipids identify antecedent memory impairment in older adults. *Nature Medicine*.

[111] Jové M., Naudí A., Gambini J. (2017). A stress-resistant lipidomic signature confers extreme longevity to humans. *The Journals of Gerontology. Series A, Biological Sciences and Medical Sciences*.

[112] Fontana L., Partridge L., Longo V. D. (2010). Extending healthy life span - from yeast to humans. *Science*.

[113] Piper M. D., Partridge L., Raubenheimer D., Simpson S. J. (2011). Dietary restriction and aging: a unifying perspective. *Cell Metabolism*.

[114] de Cabo R., Carmona-Gutierrez D., Bernier M., Hall M. N., Madeo F. (2014). The search for antiaging interventions: from elixirs to fasting regimens. *Cell*.

[115] Fontana L., Partridge L. (2015). Promoting health and longevity through diet: from model organisms to humans. *Cell*.

[116] Lee C., Longo V. (2016). Dietary restriction with and without caloric restriction for healthy aging. *F1000 Research*.

[117] Santos J., Leitão-Correia F., Sousa M. J., Leão C. (2016). Dietary restriction and nutrient balance in aging. *Oxidative Medicine and Cellular Longevity*.

[118] Eisenberg T., Knauer H., Schauer A. (2009). Induction of autophagy by spermidine promotes longevity. *Nature Cell Biology*.

[119] Hubbard B. P., Sinclair D. A. (2014). Small molecule SIRT1 activators for the treatment of aging and age-related diseases. *Trends in Pharmacological Sciences*.

[120] Madeo F., Pietrocola F., Eisenberg T., Kroemer G. (2014). Caloric restriction mimetics: towards a molecular definition. *Nature Reviews Drug Discovery*.

[121] Sinclair D. A., Guarente L. (2014). Small-molecule allosteric activators of sirtuins. *Annual Review of Pharmacology and Toxicology*.

[122] Leonov A., Arlia-Ciommo A., Piano A. (2015). Longevity extension by phytochemicals. *Molecules*.

[123] Moskalev A., Chernyagina E., de Magalhães J. P. (2015). Geroprotectors.org: a new, structured and curated database of current therapeutic interventions in aging and age-related disease. *Aging (Albany NY)*.

[124] Moskalev A., Chernyagina E., Tsvetkov V. (2016). Developing criteria for evaluation of geroprotectors as a key stage toward translation to the clinic. *Aging Cell*.

[125] Baur J. A., Pearson K. J., Price N. L. (2006). Resveratrol improves health and survival of mice on a high-calorie diet. *Nature*.

[126] Ingram D. K., Zhu M., Mamczarz J. (2006). Calorie restriction mimetics: an emerging research field. *Aging Cell*.

[127] Lane M. A., Roth G. S., Ingram D. K. (2007). Caloric restriction mimetics: a novel approach for biogerontology. *Methods in Molecular Biology*.

[128] Lutchman V., Dakik P., McAuley M. (2016). Six plant extracts delay yeast chronological aging through different signaling pathways. *Oncotarget*.

[129] Lutchman V., Medkour Y., Samson E. (2016). Discovery of plant extracts that greatly delay yeast chronological aging and have different effects on longevity-defining cellular processes. *Oncotarget*.

[130] Lefebvre P., Cariou B., Lien F., Kuipers F., Staels B. (2009). Role of bile acids and bile acid receptors in metabolic regulation. *Physiological Reviews*.

[131] Goldberg A. A., Kyryakov P., Bourque S. D., Titorenko V. I. (2010). Xenohormetic, hormetic and cytostatic selective forces driving longevity at the ecosystemic level. *Aging (Albany NY)*.

[132] Gomez-Perez A., Kyryakov P., Burstein M. T. (2016). Empirical validation of a hypothesis of the hormetic selective forces driving the evolution of longevity regulation mechanisms. *Frontiers in Genetics*.

[133] Kyryakov P., Gomez-Perez A., Glebov A. (2016). Empirical verification of evolutionary theories of aging. *Aging (Albany NY)*.

[134] Connerth M., Tatsuta T., Haag M., Klecker T., Westermann B., Langer T. (2012). Intramitochondrial transport of phosphatidic acid in yeast by a lipid transfer protein. *Science*.

[135] Aaltonen M. J., Friedman J. R., Osman C. (2016). MICOS and phospholipid transfer by Ups2-Mdm35 organize membrane lipid synthesis in mitochondria. *The Journal of Cell Biology*.

[136] Dimmer K. S., Rapaport D. (2017). Mitochondrial contact sites as platforms for phospholipid exchange. *Biochimica et Biophysica Acta*.

[137] Tatsuta T., Langer T. (2017). Intramitochondrial phospholipid trafficking. *Biochimica et Biophysica Acta*.

[138] McMahon H. T., Gallop J. L. (2005). Membrane curvature and mechanisms of dynamic cell membrane remodelling. *Nature*.

[139] Zimmerberg J. (2006). Membrane biophysics. *Current Biology*.

[140] Shibata Y., Hu J., Kozlov M. M., Rapoport T. A. (2009). Mechanisms shaping the membranes of cellular organelles. *Annual Review of Cell and Developmental Biology*.

[141] van Meer G., Voelker D. R., Feigenson G. W. (2008). Membrane lipids: where they are and how they behave. *Nature Reviews Molecular Cell Biology*.

[142] McMahon H. T., Boucrot E. (2015). Membrane curvature at a glance. *Journal of Cell Science*.

[143] Jarsch I. K., Daste F., Gallop J. L. (2016). Membrane curvature in cell biology: an integration of molecular mechanisms. *The Journal of Cell Biology*.

[144] Mårtensson C. U., Doan K. N., Becker T. (2017). Effects of lipids on mitochondrial functions. *Biochimica et Biophysica Acta*.

[145] Choi S. Y., Huang P., Jenkins G. M., Chan D. C., Schiller J., Frohman M. A. (2006). A common lipid links Mfn-mediated mitochondrial fusion and SNARE-regulated exocytosis. *Nature Cell Biology*.

[146] Huang H., Frohman M. A. (2009). Lipid signaling on the mitochondrial surface. *Biochimica et Biophysica Acta*.

[147] Osman C., Voelker D. R., Langer T. (2011). Making heads or tails of phospholipids in mitochondria. *The Journal of Cell Biology*.

[148] Yang C. Y., Frohman M. A. (2012). Mitochondria: signaling with phosphatidic acid. *The International Journal of Biochemistry & Cell Biology*.

[149] Zhang Q., Tamura Y., Roy M., Adachi Y., Iijima M., Sesaki H. (2014). Biosynthesis and roles of phospholipids in mitochondrial fusion, division and mitophagy. *Cellular and Molecular Life Sciences*.

[150] Vögtle F. N., Keller M., Taskin A. A. (2015). The fusogenic lipid phosphatidic acid promotes the biogenesis of mitochondrial outer membrane protein Ugo1. *The Journal of Cell Biology*.

[151] Giorgio M., Trinei M., Migliaccio E., Pelicci P. G. (2007). Hydrogen peroxide: a metabolic by-product or a common mediator of ageing signals?. *Nature Reviews Molecular Cell Biology*.

[152] Bonawitz N. D., Chatenay-Lapointe M., Pan Y., Shadel G. S. (2007). Reduced TOR signaling extends chronological life span via increased respiration and upregulation of mitochondrial gene expression. *Cell Metabolism*.

[153] Gems D., Partridge L. (2008). Stress-response hormesis and aging: "that which does not kill us makes us stronger". *Cell Metabolism*.

[154] Pan Y., Shadel G. S. (2009). Extension of chronological life span by reduced TOR signaling requires down-regulation of Sch9p and involves increased mitochondrial OXPHOS complex density. *Aging (Albany NY)*.

[155] Ristow M., Zarse K. (2010). How increased oxidative stress promotes longevity and metabolic health: the concept of mitochondrial hormesis (mitohormesis). *Experimental Gerontology*.

[156] Pan Y., Schroeder E. A., Ocampo A., Barrientos A., Shadel G. S. (2011). Regulation of yeast chronological life span by TORC1 via adaptive mitochondrial ROS signaling. *Cell Metabolism*.

[157] Ristow M., Schmeisser S. (2011). Extending life span by increasing oxidative stress. *Free Radical Biology and Medicine*.

[158] Barrientos A. (2012). Complementary roles of mitochondrial respiration and ROS signaling on cellular aging and longevity. *Aging (Albany NY)*.

[159] Ocampo A., Liu J., Schroeder E. A., Shadel G. S., Barrientos A. (2012). Mitochondrial respiratory thresholds regulate yeast chronological life span and its extension by caloric restriction. *Cell Metabolism*.

[160] The Saccharomyces Genome Database (SGD).

